# Microglomerular Synaptic Complexes in the Sky-Compass Network of the Honeybee Connect Parallel Pathways from the Anterior Optic Tubercle to the Central Complex

**DOI:** 10.3389/fnbeh.2016.00186

**Published:** 2016-10-07

**Authors:** Martina Held, Annuska Berz, Ronja Hensgen, Thomas S. Muenz, Christina Scholl, Wolfgang Rössler, Uwe Homberg, Keram Pfeiffer

**Affiliations:** ^1^Department of Biology, Animal Physiology, Philipps-University MarburgMarburg, Germany; ^2^Biozentrum, Behavioral Physiology and Sociobiology (Zoology II), University of WürzburgWürzburg, Germany

**Keywords:** sky-compass orientation, insect brain, central complex, polarization vision, honeybee, synaptic connections, anterior optic tubercle

## Abstract

While the ability of honeybees to navigate relying on sky-compass information has been investigated in a large number of behavioral studies, the underlying neuronal system has so far received less attention. The sky-compass pathway has recently been described from its input region, the dorsal rim area (DRA) of the compound eye, to the anterior optic tubercle (AOTU). The aim of this study is to reveal the connection from the AOTU to the central complex (CX). For this purpose, we investigated the anatomy of large microglomerular synaptic complexes in the medial and lateral bulbs (MBUs/LBUs) of the lateral complex (LX). The synaptic complexes are formed by tubercle-lateral accessory lobe neuron 1 (TuLAL1) neurons of the AOTU and GABAergic tangential neurons of the central body’s (CB) lower division (TL neurons). Both TuLAL1 and TL neurons strongly resemble neurons forming these complexes in other insect species. We further investigated the ultrastructure of these synaptic complexes using transmission electron microscopy. We found that single large presynaptic terminals of TuLAL1 neurons enclose many small profiles (SPs) of TL neurons. The synaptic connections between these neurons are established by two types of synapses: divergent dyads and divergent tetrads. Our data support the assumption that these complexes are a highly conserved feature in the insect brain and play an important role in reliable signal transmission within the sky-compass pathway.

## Introduction

Many insects have well developed abilities for orientation and navigation. En route, they rely on different strategies, like landmark navigation or vector integration (reviewed by Wehner, 2003; Menzel et al., 2006; Collett et al., 2013; Srinivasan, 2015). For spatial orientation many insects use sky-compass cues, like the position of the sun, the chromatic gradient and the polarization pattern of the sky (reviewed in Homberg et al., 2011). The ability to navigate in relation to the polarization pattern of the sky was first shown in behavioral studies on honeybees by von Frisch (1949). The neuronal basis and mechanisms underlying sky-compass orientation have been investigated anatomically and physiologically in a most detailed manner in locusts and crickets (reviewed by Homberg et al., 2011), whereas in honeybees, research into this topic is still at the beginning. Recently the sky-compass pathway in the honeybee brain has been described anatomically from the compound eye up to the lateral complex (LX; Zeller et al., 2015). The goal of this study is to investigate the anatomy of this pathway further from the LX into the central complex (CX), a neuropil which, amongst other functions, holds a neuronal representation of space around the animal (reviewed by Pfeiffer and Homberg, 2014).

The sky-compass pathway receives input via a specialized area of the compound eye, the dorsal rim area (DRA). DRA photoreceptors project through the lamina (LA) and terminate in the DRA of the medulla (MEDRA). Transmedulla neurons ramify in the MEDRA. Their fibers run dorsoventrally through the medulla (ME) and enter the lower unit complex (LUC) of the anterior optic tubercle (AOTU) via the anterior optic tract (AOT). From there two types of neuron, tubercle-lateral accessory lobe neuron 1a (TuLAL1a) and TuLAL1b project toward the LX and end in conspicuously large synaptic terminals in the lateral and the medial bulbs (LBUs, MBUs; Mota et al., 2011; Zeller et al., 2015). In the desert locust tangential TL2 and TL3 neurons of the lower division of the central body (CBL) have dendritic branches in the bulbs, forming large synaptic complexes with the terminals of TuLAL1 neurons (Vitzthum et al., 2002; Träger et al., 2008). The boundaries of the bulbs are defined by the presence of these microglomerular synaptic complexes. Locust TL2 and TL3 neurons are immunoreactive with antisera against γ-aminobutyric acid (GABA) and therefore, can be labeled using immunocytochemistry (Homberg et al., 1999). Large synaptic structures in the bulbs, either from TuLAL1 or TL neurons, have been found in other insect species as well, such as the fruit fly *Drosophila melanogaster* (Hanesch et al., 1989; Seelig and Jayaraman, 2013), the moth *Manduca sexta* (Homberg et al., 1990), the cricket *Gryllus bimaculatus* (Sakura et al., 2008), the monarch butterfly *Danaus plexippus* (Heinze and Reppert, 2011), the bumblebee *Bombus ignitus* (Pfeiffer and Kinoshita, 2012), and the desert ant *Cataglyphis fortis* (Schmitt et al., 2016). While in most of these species these neurons are involved in sky-compass vision, in *Drosophila melanogaster* a different function has been found. The dendrites of the equivalent to TL neurons, called ring neurons, represent visual features of the environment with a strong preference for a vertical stripe. The associated microglomeruli in the bulbs are arranged retinotopically and therefore form a spatial map of the visual field of the fly (Seelig and Jayaraman, 2013). Additionally, these neurons have been found to be activated by an optic flow pattern around the yaw axis (Weir and Dickinson, 2015). Thus far the sky-compass pathway of the honeybee has been traced with anatomical methods from the DRA to the bulbs of the LX (Mota et al., 2011; Zeller et al., 2015). The neurons in this pathway share many anatomical features with those of locusts, where electrophysiological studies revealed their sensitivity to polarized and chromatic light stimuli (el Jundi et al., 2014). In this study we investigate the sky-compass pathway in the honeybee from the LUC of the AOTU to the central body (CB). To reveal whether neurons from the LUC are connected to GABA-immunoreactive tangential neurons of the CB as shown in locusts, we analyzed the anatomy and ultrastructure of synaptic complexes in the MBUs and LBUs.

## Materials and Methods

### Animals

Worker honeybees (*Apis mellifera*) were caught at the entrance of the hive, which was maintained at the Department of Biology at the Philipps-University Marburg. Injections and immunostainings were performed in spring and summer, when the colony was outside. The preparations for transmission electron microscopy were made in winter. At this time the hive was kept inside a greenhouse at 25°C, and bees were fed with honey water (20–30% honey) and pollen. Experiments for synapsin/f-actin double labeling for 3D reconstructions were made during the winter season using adult worker bees (“winterbees”) from inside a colony maintained at the departmental bee station at the University of Würzburg.

### Preparation

Bees were cooled at 4°C until immobilized. For better handling during preparation, the animals were waxed to a holder with dental wax. The cuticle of the frons between the compound eyes, ocelli and labrum was removed. For getting access to the brain, the hypopharyngeal glands and air-sacks as well as the neural sheath were removed.

### Extracellular Iontophoretic Dye Injection

Extracellular iontophoretic dye injections were performed to achieve staining of small numbers of neurons (1–20) connecting the AOTU to the bulbs of the LX or the bulbs to the CX. Sharp glass microelectrodes were fabricated by pulling borosilicate capillary tubes (outer diameter 1.5 mm, inner diameter 0.75 mm, Hilgenberg, Malsfeld, Germany) with a Flaming/Brown puller (P97, Sutter instrument, Novato, CA, USA). Electrode tips were filled with 4% Neurobiotin tracer (Vector Laboratories, Burlingame, CA, USA) in 1 M KCl and backed up with 2.5 M KCl. These electrodes had a resistance of 100–200 MΩ in the tissue. Using a micromanipulator an electrode was positioned in the area of the LUC of the AOTU or the CBL. By applying a pulsed current of 10 nA (1 Hz, 50% duty cycle) for 20–45 min the tracer was ejected from the electrode and entered the neurons in the vicinity of the tip presumably through pores created by an electroporating effect of the electric field. After removing the electrode, brains were dissected from the head capsule and immersed in a fixative containing 4% paraformaldehyde (Sigma-Aldrich, Steinheim, Germany), 0.25% glutaraldehyde (Carl Roth, Karlsruhe, Germany) and 0.25% saturated picric acid in 0.1 M phosphate buffered saline (PBS, pH 7.4) overnight at 4°C. They were then washed with PBS. To detect neurons labeled with Neurobiotin, brains were immersed in a solution containing Cy3-conjugated streptavidin (1:1000, Jackson Immunoresearch Laboratories, West Grove, PA, USA), 0.3% Triton X-100 (TrX; Sigma, Deisenhofen, Germany) and PBS. After incubation at 4°C for 3 days, brains were washed with PBS and 0.3% TrX (PBT) and afterwards with PBS. The brains were then dehydrated in an ascending ethanol series. To increase image quality brains were cleared with methyl salicylate (Merck, Darmstadt, Germany). Finally, the brains were embedded between two cover slips in Permount (Fisher Scientific, Pittsburgh, PA, USA). Eight reinforcement rings (Zweckform, Oberlaindern, Germany) served as spacers to prevent squishing the tissue.

### Mass Staining Procedure

For tracing of TuLAL1 neurons, dextran Texas Red crystals (lysine-fixable, 3000 MW, Molecular Probes, Eugene, OR, USA) were inserted into the LUC. To do this, the tip of a sharp glass microcapillary, that was created as described above, was broken to a diameter of about 5–30 μm. The tip was dipped into petroleum jelly and then into the dextran Texas Red to pick up a few tracer crystals. After removing all liquid around the brain with a piece of paper tissue, the microcapillary was manually advanced into the target area. Excess dye was washed off with Ringer solution (130 mM NaCl, 5 mM KCl, 4 mM MgCl_2_, 15 mM HEPES, 25 mM glucose, 160 mM saccharose, 5 mM CaCl_2_). To allow for complete uptake and distribution of the tracer in the neurons, the head capsule was covered with tissue paper and the bee was kept overnight at 4°C in a moist chamber. To prevent bleaching of the fluorescent dye all further steps were performed in darkness if possible. After removing the brain from the head capsule it was fixed overnight at 4°C in 4% paraformaldehyde and 0.5% glutaraldehyde in 0.1 M sodium phosphate buffer (NaPi; pH 7.4). The brain was then washed with 0.01 M PBS. After embedding in albumin-gelatin (12% ovalbumin and 4.8% gelatin in demineralized water) and fixation overnight at 4°C with 8% formaldehyde in NaPi, the brain was sectioned at 40 μm in the frontal plane using a vibrating-blade microtome (VT 1000S or VT 1200S; Leica, Wetzlar, Germany).

### GABA Immunostaining

To label GABA-immunoreactive neurons, we used two different antisera that were raised against GABA conjugated to keyhole-limpet hemocyanin (KLH) via glutaraldehyde. The first antiserum was raised in guinea pig (ab17413; Lot GR51659; Abcam, Cambridge, UK). According to the manufacturer the specificity of the antiserum was tested on brain slices of rats by preadsorption with 100 nM GABA conjugated to glutaraldehyde, which abolished all staining. Preadsorption with 500 nM of similar conjugates of glutamic acid, glutamate and taurine failed to block staining (product datasheet anti-GABA antibody ab17413). The second antibody was raised in rabbit (# 9/24; kindly provided by Dr. T.G. Kingan). It had been affinity purified against KLH. The specificity of this antiserum was tested on brain sections of the sphinx moth *Manduca sexta*, the honeybee and the desert locust *Schistocerca gregaria*. In *Manduca sexta* liquid-phase preadsorption of the diluted antiserum with GABA-glutaraldehyde-KLH and similar conjugates of L-glutamic acid, β-alanine, L-glutamine and taurine was performed (Hoskins et al., 1986). GABA-glutaraldehyde-KLH blocked immunostaining at a concentration of 24 nM, whereas similar concentrations of the other amino acid conjugates were without effect (Hoskins et al., 1986). Likewise, on brain sections of the honeybee, preadsorption with 1 mM GABA-glutaraldehyde completely blocked labeling (Schäfer and Bicker, 1986), and in the desert locust, preadsorption with 15 nM GABA-glutaraldehyde-bovine serum albumin (BSA) conjugate abolished all staining on brain sections (Homberg et al., 1999).

For double staining of tracer-injected brains with GABA antiserum, gelatin slices were washed with 0.1% TrX in saline substituted Tris-buffer (SST; pH 7.4). Sodium borohydride was used to reduce background autofluorescence caused by Schiff bases that occur during glutaraldehyde fixation (Baschong et al., 1999). Sections were covered for 10 min with 10 mg/ml NaBH_4_ and 0.1% TrX in NaPi. Deposit was washed out with 0.1% TrX in SST. To block unspecific binding sites the slices were pre-incubated for 1 h at room temperature on a shaker with 10% normal donkey serum (NDS; Dianova, Hamburg, Germany), 0.5% TrX and SST. The primary antibody against GABA was diluted 1:500 in a solution of 1% NDS, 0.02% sodium azide and 0.5% TrX in SST. Slices were incubated overnight at 30°C in an incubator on a shaker. After washing in SST containing 0.1% TrX, sections were treated with the secondary antibody solution. It consisted of Cy2-conjugated donkey anti-guinea pig IgG against the antiserum from Abcam (1:300; Dianova, Hamburg, Germany) and donkey anti-rabbit IgG against the antiserum from Kingan (1:200; Dianova, Hamburg, Germany), 1% NDS and 0.5% TrX in SST. The secondary antiserum was applied for 1 h on a shaker at room temperature. After further washing with 0.1% TrX in SST the sections were mounted on chromalum/gelatin-coated microscope slides, dehydrated in an ascending ethanol series, and embedded in Entellan (Merck, Darmstadt, Germany) under cover slips.

### F-actin Staining and Immunolabeling for Synapsin

To obtain an overview of all synaptic complexes in the bulbs of the LX, we performed double labeling for the vesicle-associated protein synapsin and filamentous actin (see Groh et al., 2004; Schmitt et al., 2016). Brains were dissected from the head capsule and immediately fixed with ice-cold 4% paraformaldehyde (methanol free, 28908, Fischer Scientific, Schwerte, Germany) in PBS overnight at 4°C. After washing with PBS, brains were embedded in 5% low-melting point agarose (Agarose II, no. 210–815, Amresco, Solon, OH, USA), adjusted to a frontal plane and sectioned at 100 μm thickness using a vibrating-blade microtome (VT 1000S; Leica, Wetzlar, Germany). Preincubation was performed using 0.2% TrX in PBS containing 2% normal goat serum (NGS, 005-000-121, Jackson ImmunoResearch Laboratories, West Grove, PA, USA) for 1 h at room temperature. To visualize f-actin, sections were incubated with 0.2 units Alexa Fluor 488 conjugated phalloidin (A12379, Molecular Probes, Eugene, OR, USA) in 0.2% TrX and 2% NGS in PBS for 3 days at 4°C. For the additional labeling of synapsin, a monoclonal antibody raised against the *Drosophila* synaptic-vesicle-associated protein synapsin I (SYNORF1, kindly provided by E. Buchner, University of Würzburg, Germany) was added (1:50). SYNORF1 in honeybee tissue has been characterized by Pasch et al. (2011). Sections were washed several times in PBS, before incubated in Alexa Fluor 568 conjugated goat anti-mouse (1:250, A11004, Molecular Probes, Eugene, OR, USA) in PBS with 1% NGS for 2 h at room temperature. After washing with PBS, sections were transferred into 60% glycerol in PBS for 30 min. They were then mounted in 80% glycerol in PBS on glass slides covered with cover slips.

### Wholemount Preparation for Neuropil Reconstruction

Brains were dissected from the head capsule as described above and fixed with ice-cold 2% paraformaldehyde and 2% glutaraldehyde in PBS for 4 days at 4°C. After several washing steps with PBS the brain tissue was dehydrated in an ascending ethanol series (50%, 70%, 90%, 95% and 3 × 100% for 10 min each) before being cleared in methyl salicylate for 4 days at 4°C. Brains were then mounted in methyl salicylate in custom metal slides covered with cover slips (method adapted from Kuebler et al., 2010).

### Transmission Electron Microscopy

To investigate the ultrastructure of synaptic complexes brains were fixed using the high-pressure freezing technique (McDonald, 2007; Müller-Reichert et al., 2007; Rachel et al., 2010; Peschke et al., 2013). Dissected brains were prefixed overnight at 4°C with 4% paraformaldehyde and 2.5% glutaraldehyde in 0.1 M sodium cacodylate buffer (NaCB; pH 7.2). After washing in 0.1 M NaCB, brains were embedded in 7% low-melting point agarose (LM3, AppliChem GmbH, Darmstadt, Germany), and a 200 μm thick slice, containing the area of interest, was cut with a vibratome. These sections were then high-pressure frozen with a Wohlwend HPF Compact 02 (M. Wohlwend, Engineering Office, Sennwald, Switzerland). They were then transferred to an automatic ASF2 freeze substitution unit (Leica Microsystems, Wetzlar, Germany) to replace the water and enhance contrast. For cryo-substitution fixation (CSF) a solution of 0.2% OsO_4_, 0.25% uranyl acetate and 5% (vol/vol) H_2_O in acetone (A.O.U.H; Walther and Ziegler, 2002; Junglas et al., 2008; Rachel et al., 2010) was added. Freeze-substitution was carried out at −90°C for 46.5 h, −60°C for 8 h, −30°C for 8 h and held at 0°C for 3 h. The heating time between the steps was 1 h. Afterwards, the sections were washed twice with ice-cold acetone (100%) and were then gradually infiltrated with Epon at room temperature, followed by polymerization for 72 h at 60°C. Ultrathin sections (60–80 nm) were cut with an ultramicrotome (Ultracut; Reichert-Labtech, Wolfratshausen, Germany), collected on uncoated copper 400 mesh grids (Plano, Wetzlar, Germany) and contrast enhanced by positive staining with 2% uranyl acetate and lead citrate (Reynolds, 1963).

### Image Acquisition and Processing

Images of fluorescent samples were acquired with a confocal laser scanning microscope (CLSM; TCS SP5 and TCS SP2, Leica Microsystems, Wetzlar, Germany). Optical serial sections of an overview of all synaptic complexes in the tracer-injected brains immunostained for GABA were scanned using a 40× objective (HCX PL APO 40×/1.25−0.75 Oil Lbd. bl.; Leica, Bensheim. Germany) at a resolution of 1024 × 1024 pixels and a *z*-stepsize of 1.5 μm. For detailed scans at the same resolution with a *z*-stepsize of 1 μm a 63× objective (HCX PL APO 63×/1.3 GLY CORR CS 21, Leica, Bensheim, Germany) was used. For double labeled synapsin/f-actin preparations, physical sections containing the whole two clusters of synaptic complexes in the bulbs were selected and scanned at a resolution of 1024 × 1024 pixels using a 20× objective (HC PL APO 20×/0.70 Imm, Leica, Bensheim, Germany) and 63× objective (HCX PL APO 63×/1.4−0.6 Oil, Leica, Bensheim, Germany) to obtain image stacks at a *z*-stepsize of 1 μm. Exploiting the increased autofluorescence attributes of the paraformaldehyde/glutaraldehyde-fixed wholemount preparations, these brains were scanned with a 10× objective (HC PL APO 10×/0.4 Imm, Leica, Bensheim, Germany) at a *z*-stepsize of 4 μm in three tiles to create a panoramic overview image stack of the whole brain.

All image stacks were processed with Amira (versions 3.1.1 and 5.3.3; FEI Visualization Sciences Group, Mérignac Cedex, France). Amira was further used for the 3D reconstruction of individual synaptic complexes in the bulbs based on f-actin positive profiles in synapsin/f-actin double labeled preparations and to create a whole brain reconstruction of all major neuropils based on autofluorescence wholemount preparations. To evaluate the spatial distribution and localization of synaptic complexes in the context of the whole brain the synaptic reconstructions were transformed into the whole brain reconstruction using the CX as a landmark for orientation. Volumes of the reconstructed postsynaptic portion of the microglomeruli were calculated using Amira 5.6. The data for the lateral and medial cluster were statistically compared using the Mann-Whitney test (VassarStats^1^).

Transmission electron micrographs were taken using a JEOL JEM-2100 transmission electron microscope (JEOL, Tokio, Japan) at an acceleration voltage of 120 kV. Images were taken with a 2k × 2k pixel CCD-camera F214 and the software EM-Menu 4 (TVIPS, Gauting, Germany). Contrast and brightness were optimized with Adobe Photoshop CC (Adobe Systems, San Jose, CA, USA) software if necessary, and all figures were created with Adobe Illustrator CC.

## Results

**Figure 1:**
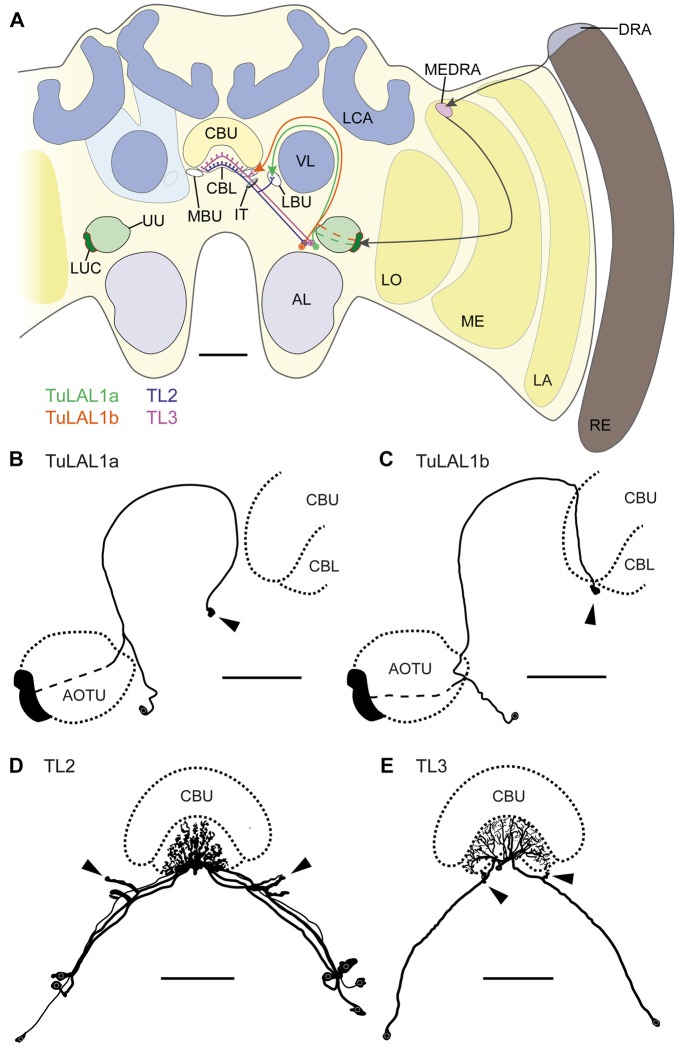
**(A)** Schematic drawing of the position of the neuron types forming microglomerular synaptic complexes in the medial and lateral bulbs of the honeybee *Apis mellifera*. The connection from the anterior optic tubercle to the central complex (CX) is formed by tubercle-lateral accessory lobe neurons 1a (TuLAL1a; green) and TuLAL1b neurons (orange). Two types of tangential neuron (TL2, blue; TL3, purple) provide input into the lower division of the central body (CBL). **(B–E)** Reconstructed morphologies of neurons labeled by extracellular dye injections. TuLAL1a **(B)** and TuLAL1b **(C)** neurons have their cell bodies medially from the AOTU. The axons of both types run toward the central body (CB), where they end in large terminals (arrowheads). In TuLAL1a neurons these terminals are located in the LBU (**B**, arrowhead), whereas TuLAL1b neurons terminate in the MBU (**C**, arrowhead), close to the CBL. TL neurons have their cell bodies medially to the AOTU and posteriorly to the somata of TuLAL1 neurons. Their primary neurites run toward the isthmus tract (IT), where they give off sidebranches. The sidebranches of TL2 neurons extend into the LBU, and those of TL3 to the MBU (**D,E**, arrowheads). The axons extend from the bulbs further into the CBL, where they branch in all slices but not in all layers. TL2 neurons branch in the dorsal part of the CBL, TL3 neurons in the ventral part. AL, antennal lobe; CBU, upper division of the CB; DRA, dorsal rim area; LA, lamina; LBU, lateral bulb; LCA, lateral calyx; LO, lobula; LUC, lower unit complex of the AOTU; MBU, medial bulb; ME, medulla; MEDRA, dorsal rim area of the medulla; RE, retina; UU, upper unit of the AOTU; VL, vertical lobe. Scale bars: **A** = 200 μm, **B–E** = 100 μm.

We investigated the anatomy and ultrastructure of microglomerular synaptic complexes in the bulbs of the LX that connect the AOTU to the CB in the brain of the honeybee *Apis mellifera* (Figure 1). We first describe the different neuron types that are involved in these conspicuous connections. Then the general distribution and appearance of the synaptic complexes is shown. Last, we present data on the subcellular organization and show two types of synapses forming cell-cell connections. Positional information within the brain is given with respect to the body axis. For neuropils we followed the terminology suggested by Ito et al. (2014) wherever possible. Additionally, we refer to the entirety of small subunits of the AOTU as “LUC” as suggested by Zeller et al. (2015). The nomenclature of all neurons corresponds to the terminology used in locusts, monarch butterflies and bumblebees (Müller et al., 1997; Homberg et al., 2003; Pfeiffer et al., 2005; Heinze and Reppert, 2011; Pfeiffer and Kinoshita, 2012).

### Neurons Innervating the Bulbs

Using extracellular iontophoretic dye injections, we were able to identify and distinguish two subtypes of TuLAL1 neuron connecting the LUC of the AOTU to the bulbs. The bulbs are neuropils located laterally on both sides of the CX. In each hemisphere there are two bulbs: the MBU and LBU. Together with the lateral accessory lobe they form the LX which is closely associated with the CX (Ito et al., 2014). Both subtypes of TuLAL1 neuron had their cell bodies medially to the AOTU and their axons extended around the vertical lobe (VL) of the mushroom body toward the CX. The axons of both subtypes ended in conspicuous large, hat-like terminals. The majority of the injected neurons had only one of those synaptic endings, but in a few cases the axon ended in more than one terminal. In these cases, the terminals were always in close proximity to each other and in the same bulb. The terminals of TuLAL1a neurons were located in the LBU, ventrolaterally to the CB (Figure 1B). The second subtype, TuLAL1b neurons, projected to the MBU which lies directly adjacent to the groove formed between the lateral boundary of the lower and upper divisions of the CB (Figure 1C). The transmission of information from the bulbs into the CB is assumed to take place in tangential neurons of the type TL2 and TL3, the presumptive equivalent to ring neurons in the fruit fly (*Schistocerca gregaria*: Vitzthum et al., 2002; Träger et al., 2008; *Drosophila melanogaster*: Seelig and Jayaraman, 2013; Wolff et al., 2015). We were able to identify these neuronal cell types in the honeybee brain. They had their cell bodies medially to the AOTU and posteriorly from the somata of TuLAL1 neurons. Their primary neurites ran toward the isthmus tract (IT), where they gave off single large side branches that extended into one of the bulbs and had dense accumulations of protrusions at their tips (Figures 1D,E arrowheads). These dendritic side branches were about 20 μm to 50 μm long in TL2 neurons (Figure 1D) and invaded the LBU. In contrast, in TL3 neurons they had a stub-like appearance of only a few μm and innervated the MBU (Figure 1E). The axons of both types of neuron extended into the CBL, where they branched in all slices of defined layers.

Owing to the close morphological similarity of these four neuron types with equivalent neurons in locusts, monarch butterflies and bumblebees (Müller et al., 1997; Homberg et al., 2003; Pfeiffer et al., 2005; Heinze and Reppert, 2011; Pfeiffer and Kinoshita, 2012), we suppose that these neurons form the large synaptic complexes found in the bulbs. More precisely, TuLAL1a neurons develop synaptic connections with TL2 and TuLAL1b with TL3 neurons (Figure 1A).

### Appearance of the Microglomerular Synaptic Complexes

**Figure 2:**
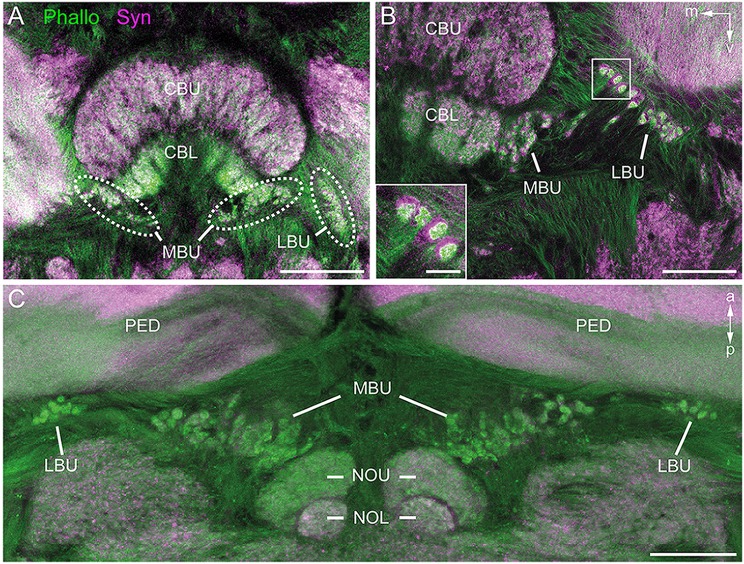
**Anti-synapsin (Syn, magenta) and f-actin phalloidin (Phallo, green) staining of the microglomerular synaptic complexes. (A)** Frontal sections show that the synaptic complexes are arranged in two clusters: one in the MBU close to the connection of the CBL and the upper division (CBU). The second cluster is located in the LBU. **(B)** At higher magnification of both clusters the distribution of the anti-synapsin and f-actin phalloidin staining reveals a synapsin-positive cup-shaped structure with an f-actin containing profile in the center. **(C)** In horizontal sections the distribution in the anterior-posterior axis becomes apparent. The synaptic complexes in both bulbs appear posterior to the pedunculi (PED). Those of the MBU extend posterior to the upper division of the noduli (NOU). a, anterior; m, medial; NOL, lower division of the noduli; p, posterior; v, ventral. Scale bars: **A** = 100 μm, **B,C** = 50 μm, inset in **B** = 10 μm.

To obtain an overview of the synaptic complexes within the bulbs, we performed double labeling experiments using anti-synapsin and f-actin phalloidin staining. Synaptic complexes were clearly visible using this method and arranged in two distinct clusters, one group of synaptic complexes very close to the CBL in the MBU, and another group located more laterally in the LBU (Figure 2A). Higher magnification revealed that synapsin-immunoreactivity (IR) was localized within a cup-shaped profile, and dense f-actin phalloidin staining was concentrated inside the halo of synapsin-IR (Figure 2B). The anterior-posterior expansion of both clusters becomes obvious in horizontal sections (Figure 2C).

**Figure 3:**
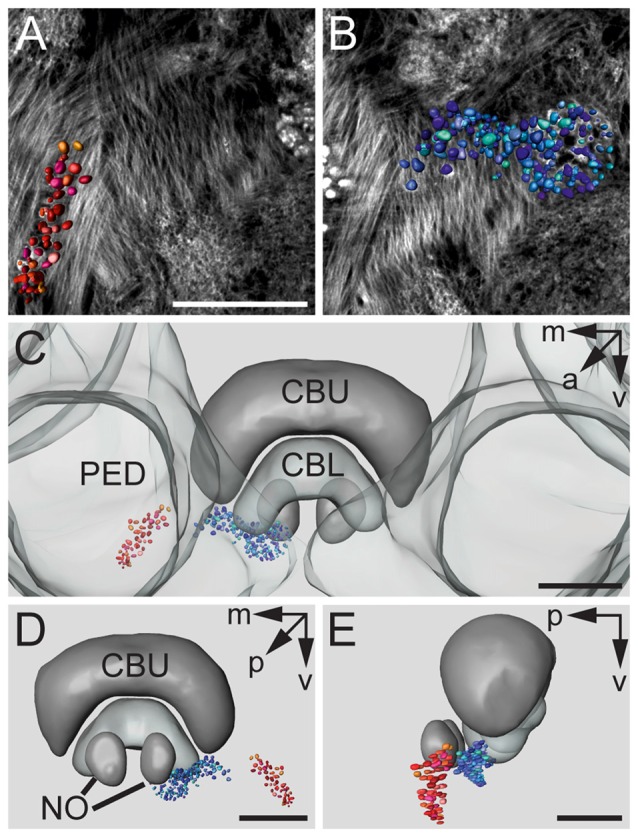
**3D reconstructions of f-actin phalloidin labeled microglomerular synaptic complexes. (A)** Reconstruction of the cluster (red) in the LBU. **(B)** Reconstruction of the synaptic complexes in the MBU (blue). **(C)** 3D reconstruction of the microglomerular synaptic complexes and their spatial distribution in relation to the PED and the CBU and CBL. **(D)** View from posterior reveals the distribution of the clusters in relation to the CB and the noduli (NO). **(E)** Sagittal view shows the location of the clusters in anterior-posterior axis extending to the NO. Scale bars: **A,B** = 50 μm, **C–E** = 70 μm.

**Figure 4:**
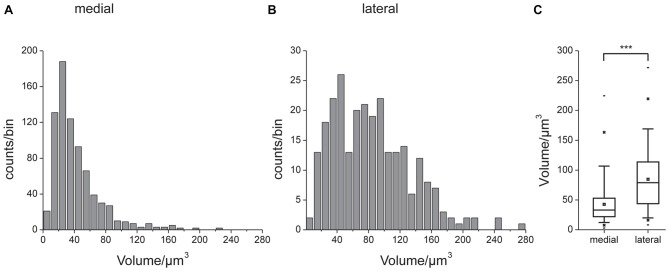
**Volumes of the reconstructed f-actin positive postsynaptic portion of the microglomeruli.** Unilateral data from four brains. Histograms of data from the medial **(A)** and lateral **(B)** cluster. Note different scaling in **(A,B)**. **(C)** Comparison of volume data. Box: 25th, 50th and 75th percentile, small square: average, whiskers: 5th and 95th percentile, cross: 1st and 99th percentile, dash: minimum and maximum value. The median microglomerulus volume in the medial cluster was 33 μm^3^, whereas it was 79 μm^3^ in the lateral cluster. This difference was statistically significant (indicated by ***, Mann-Whitney test, *P* < 0.0001, *U* = 157060.5, *z* = −13.39, *n* = 4, *N*_medial_ = 772, *N*_lateral_ = 262).

Figures 3A,B show a complete 3D reconstruction of the lateral (red) and medial (blue) clusters of one brain hemisphere of synaptic complexes merged into a tissue section of phalloidin-labeled fiber bundles. The total number of synaptic complexes within each of the two clusters was assessed by individual 3D reconstructions of phalloidin-labeled profiles revealing 68 ± 1.9 SD (*n* = 4) complexes in the lateral cluster and 197.5 ± 37.5 SD (*n* = 4) in the medial cluster (Figures 3C–E). Because synapsin-positive presynaptic profiles often appeared fused, we used the more distinct phalloidin-labeled profiles to quantify individual synaptic complexes. It cannot be excluded, however, that in some cases, particularly in the medial cluster, more than one phalloidin-labeled profile was associated with one (fused) synapsin-positive complex at this level of resolution. In addition to the total numbers of synaptic complexes defined by phalloidin-labeled clusters, the 3D reconstructions illustrate the position and extension of the two clusters in relation to other brain structures, in particular the CB. Based on the 3D reconstruction of the f-actin positive (postsynaptic) portion of the microglomeruli, we measured their volumes (Figure 4). Owing to the rather small size of these structures, compared to the *z*-resolution of the image stacks, these values should be treated with caution and should not be taken as absolute measurements. However, they illustrate the size difference between the elements of the lateral and the medial cluster as well as the distribution of volumes within the clusters. The median volume of the postsynaptic elements from four brains in the medial cluster was 33 μm^3^, whereas it was 79 μm^3^ in the lateral cluster. This difference was statistically significant (Mann-Whitney test, *P* < 0.0001, *U* = 157060.5, *z* = −13.39, *N*_medial_ = 772, *N*_lateral_ = 262). The total volume of all postsynaptic elements was 8485 μm^3^ in the medial cluster and 6244 μm^3^ in the lateral cluster (median values, *n* = 4).

**Figure 5:**
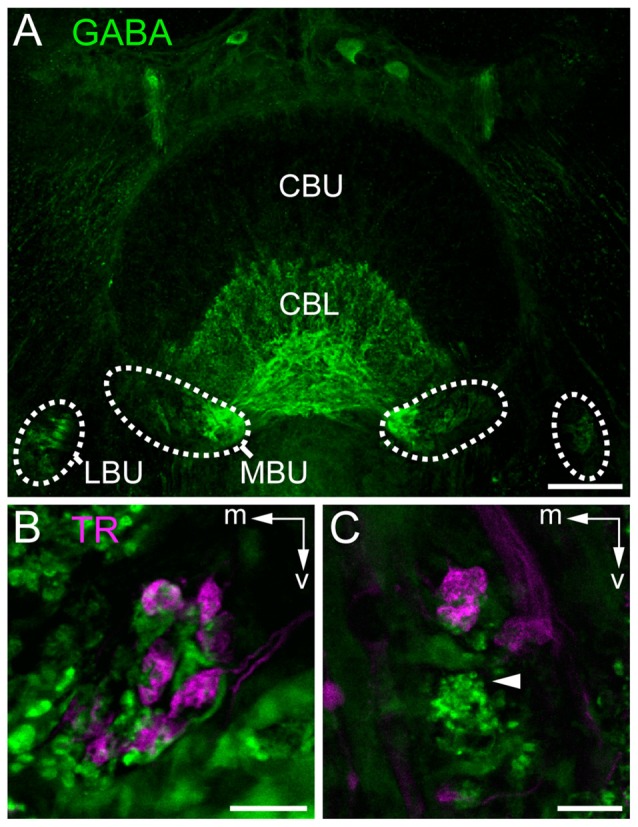
**Staining of neurons contributing to terminals in the bulbs. (A)** Immunostaining for γ-aminobutyric acid (GABA; green) using the antibody from Abcam. GABA-immunoreactive TL neurons branch in the LBUs and the larger MBUs. The neurons have dense ramifications in the CBL whereas the upper division (CBU) is barely stained. **(B,C)** Double labeling by injection of dextran Texas Red into the AOTU, labeling TuLAL1 neurons (magenta) and immunostaining for GABA (green) with the antibody of Kingan, labeling TL neurons, reveals the structure of microglomerular synaptic complexes. **(B)** In the MBU TL3 neurons form complexes with large terminals of TuLAL1b neurons. The TuLAL1b neuron terminals are located on top of the TL neuron branches. **(C)** In the LBU the complexes have a similar structure. Here, GABA immunostaining exposes a bushy structure at the tip of the extension of a TL neuron (arrowhead). Scale bars: **A** = 50 μm, **B,C** = 10 μm.

To investigate whether the synaptic complexes are formed by TuLAL1a/b and TL2/3 neurons we performed double label experiments. In *Schistocerca gregaria*, TL2 and TL3 tangential neurons of the CB are GABA-immunoreactive (Homberg et al., 1999; Träger et al., 2008). In honeybees immunostaining for GABA also labels putative tangential neurons in the CBL (Schäfer and Bicker, 1986). Immunofluorescent labeling for GABA confirmed the data of Schäfer and Bicker (1986) and revealed a subdivision of terminals of the labeled TL neurons lateral to the CB into two larger medial groups and two smaller lateral groups (Figure 5A). It also confirmed that branching of these neurons in the CB is restricted to the CBL. The few fibers stained in the upper division of the central body (CBU) likely belong to TU1 and TU2 neurons as described in *Schistocerca gregaria* (Homberg et al., 1999). To analyze whether the TL neurons are candidates for postsynaptic partners of TuLAL1 neurons, we stained TuLAL1 neurons through tracer injection into the LUC of the AOTU, followed by marking of TL neurons through GABA immunofluorescence labeling. Terminals of TuLAL1 neurons were large hat-like structures with an uneven surface (Figures 5B,C). Close inspection of single complexes in double labeled samples revealed a distinct pattern: hat-like terminals from TuLAL1 neurons partly enclosed the terminals of side branches of TL neurons (Figures 5B,C).

### Ultrastructure and Synaptic Connections

**Figure 6:**
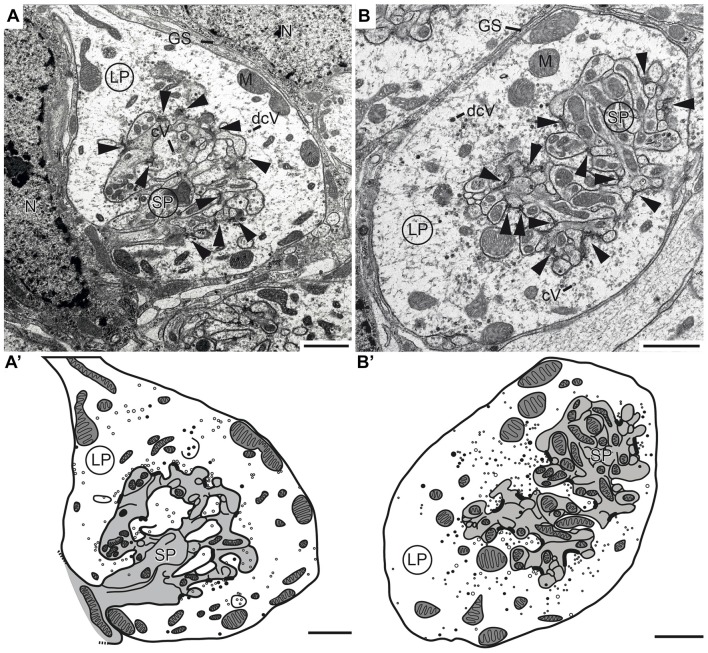
**Transmission electron micrographs showing the ultrastructure of microglomerular synaptic complexes in the LBU (A,A’) and the MBU (B,B’) of the lateral complex (LX). (A)** The complex consists of one large profile (LP) enclosing many small profiles (SPs). The LP contains clear vesicles (cVs), some large dense core vesicles (dcVs) and many mitochondria (M) and forms numerous synaptic connections with the SP (arrowheads). A glial sheath (GS) is wrapped around the complex. It is located in proximity to two nuclei of other cells (N). **(A’)** Drawing of the complex in **(A)** shows the borders of the profiles, organelles and synaptic connections. All parts of the LP are shown in white and the SP in gray. **(B,B’)** The structure of the complex in the LBU is similar to the one in the MBU: one LP encloses many SP. Scale bars = 1 μm.

To investigate the synaptic connectivity between TuLAL1 and TL neurons, we studied the microglomeruli at the ultrastructural level. Transmission electron micrographs showed that the synaptic complexes have a diameter of up to 8 μm and are partly enwrapped by layers of glia (Figures 6A,B). Therefore, individual synaptic complexes were clearly distinguishable from one another. Each microglomerular complex consisted of a single large cup-shaped profile, apparently from a TuLAL1 neuron, enclosing numerous small profiles (SPs), apparently originating from TL neurons. The large profiles (LPs) of TuLAL1 neurons were less electron dense than the small central profiles. They contained many mitochondria and two types of vesicle, numerous clear vesicles (cVs) with a diameter of 20–60 nm and a small number of dense core vesicles (dcVs) with a diameter of 50–80 nm (Figure 6). The bulk of vesicles was concentrated close to the internal membrane of the cup-shaped LP. The synaptic endings of the TL neurons formed many SP surrounded by the single LP. Apparently, one or a few processes from TL neurons enter the microglomerulus and give rise to a dense bush of ramifications in the center (Figure 6). These profiles also contained some mitochondria but additional organelles were difficult to distinguish. All synaptic connections were made at the inside of the complexes; we never found active zones at the exterior membrane of the LP.

**Figure 7:**
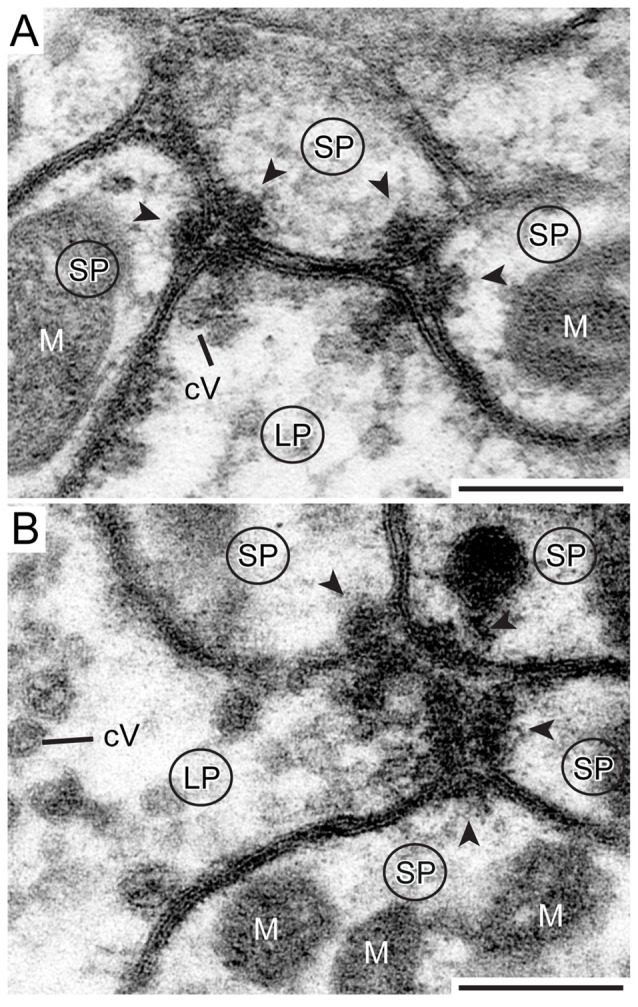
**Detailed view of two types of synapse. (A)** One presynaptic LP of a TuLAL1 neuron (LP) gives input into three small postsynaptic profiles (SP) of a TL neuron via two divergent dyads. The structure of both dyads is similar: some small cVs are near an electron dense stalk-like structure at the presynaptic membrane. The synaptic cleft between the pre- and postsynaptic membranes has a triangular structure. In both postsynaptic profiles the membranes form electron dense foldings due to transmitter receptors (arrowheads) and contain mitochondria (M). **(B)** One presynaptic terminal (LP) and four postsynaptic profiles (SP) forming a divergent tetrad. Many small cVs are concentrated at the presynaptic membrane. Scale bars = 200 nm.

Synaptic release sites were identified based on their electron dense ultrastructural specializations as described previously in other studies (Gray, 1959; Uchizono, 1965; Aghajanian and Bloom, 1967; Colonnier, 1968; Mayhew, 1996; Watson and Schürmann, 2002). The synapses we found were only formed between LPs and SPs, no synaptic connections were found between SPs. The electron dense synaptic release sites enabled us to identify the LPs of TuLAL1 neurons as presynaptic terminals. They included transmitter-containing vesicles and a number of mitochondria as described above. Additionally, an electron-dense membrane structure was present as transmitter release site. The associated membranes of the small postsynaptic profiles of TL neurons showed an electron-dense thickening, implying postsynaptic densities. Another feature of synaptic sites was a cleft of diverse thickness between the pre- and postsynaptic membranes. We were able to distinguish two types of synapses. The more frequent type was a divergent dyad where one presynaptic profile (Figure 7A, LP) was connected to two postsynaptic partners (Figure 7A, arrowheads). Due to the triangular arrangement and the preserved membranes the synaptic cleft was well defined. The presynaptic membranes showed aggregations of cVs in the vicinity of the electron-dense region. All involved postsynaptic profiles showed characteristic electron-dense membrane regions. The inside of the postsynaptic profiles was devoid of synaptic vesicles but contained mitochondria. The second type of synapse was a divergent tetrad, where the presynaptic profile (Figure 7B, LP) formed one synapse with four postsynaptic profiles (Figure 7B, arrowheads). The structure was nearly the same as in dyads: the presynaptic membrane showed an electron-dense fusion region with adjacent cVs, and a visible thickening of the postsynaptic membranes.

## Discussion

We characterized the anatomy and ultrastructure of microglomerular synaptic complexes in the bulbs of the honeybee brain. These complexes have been investigated previously in the sky-compass pathway of the locust *Schistocerca gregaria* (Träger et al., 2008) and in the brain of the desert ant *Cataglyphis fortis* (Schmitt et al., 2016), and therefore, seem to be a highly conserved feature of the insect brain. The sky-compass pathway in locusts originates in specialized photoreceptors of the DRA of the compound eye and runs through the ME toward the LUC of the AOTU and from there to the LX and into the CB (reviewed by Pfeiffer and Homberg, 2014). This pathway has been characterized anatomically in the honeybee from the DRA to the LX, and the involved neurons strongly resemble those described in the locust (Zeller et al., 2015). In this study, we focused on the synaptic contacts in the bulbs of the LX, connecting the LUC of the AOTU to the CBL. In the locust (Träger et al., 2008) and desert ant (Schmitt et al., 2016) these microglomeruli have a remarkable size and structure. Therefore, they are likely to play an important role in the processing of visual information, like sky-compass cues or visual detection, by providing proper visual input into the CX.

### Structure of the Microglomeruli of the LX Compared to Other Species

The large microglomerular synaptic complexes in *Apis mellifera* are located in the MBU and LBU of the LX. Each synaptic complex consists of one large presynaptic terminal formed by a TuLAL1a or TuLAL1b projection neuron from the LUC of the AOTU (Zeller et al., 2015). These terminals have been mentioned in a previous study (Mota et al., 2011) but have not been investigated in bees any further. The TuLAL1 neurons are connected to GABA-immunoreactive TL2 and TL3 tangential neurons of the CBL. Those TL neurons have conspicuous dendritic endings with single bushy structures at the tip of a stalk. At the ultrastructural level these endings appear as SP that are enclosed by a single LP of a TuLAL1 neuron. The large presynaptic terminals contain two types of vesicles: cVs and dcVs, but no information exists on their transmitter content. Single complexes are enclosed by glia cells. This glia can be referred to as astrocyte-like, as described in the fruit fly (Awasaki et al., 2008). It is the only known type of glia located within neuropils and associated with synaptic connections. Its function is likely the support of neurons, which in our case is very crucial considering the size and the amount of mitochondria in pre- and postsynaptic profiles. Additionally, this type of glia probably takes part in the modulation of neural connections (Awasaki et al., 2008; Edwards and Meinertzhagen, 2010).

Homologs of the involved neuron types were characterized anatomically and physiologically in many other insect species and, therefore, seem to be highly conserved. In the fruit fly *Drosophila melanogaster* GABAergic ring neurons of the ellipsoid body are homologous to TL neurons in honeybees. They form microglomeruli in the bulbs and connect them to the ellipsoid body, the equivalent of the CBL in the honeybee (Hanesch et al., 1989). Calcium-imaging experiments in tethered fruit flies showed that these microglomeruli are sensitive to visual features with an orientation tuning to vertical stripes (Seelig and Jayaraman, 2013; Weir and Dickinson, 2015). In the cricket *Gryllus bimaculatus* compass-neuron like cells (homologs of TL2 neurons in other species) that connect the LX with the CBL are sensitive to polarized light (Sakura et al., 2008). In the monarch butterfly (*Danaus plexippus*) there is only one cluster, the LBU, but different subtypes of TuLAL1 neuron ramify in spatially segregated areas. That is suggestive for a similar connectivity specificity as in honeybees. Colabeling of TL3- and TuLAL1 neurons revealed spatial proximity of large terminals of TuLAL1 neurons and profiles of TL3 neurons (Heinze and Reppert, 2011, 2012; Heinze et al., 2013). In the bumblebee *Bombus ignitus* TuLAL1a/b neurons share a very similar anatomy to the two cell types shown here (Pfeiffer and Kinoshita, 2012).

Although in all of these species one or both types of TuLAL1- and TL neuron have been described morphologically and partly investigated physiologically, the synaptic complexes they form have been explored only in desert ants and desert locusts. In the desert ant *Cataglyphis fortis* the microglomerular synaptic complexes are clustered in a single bulb (LBU; Schmitt et al., 2016) whereas in honeybees we found two clusters, one in the LBU and the other one in the MBU. Although the general anatomy appears very similar in honeybees and ants, a closer look reveals some distinct differences. In honeybees the complexes have a diameter of up to 8 μm compared to only 5 μm in ants (Schmitt et al., 2016). Likewise, the presynaptic terminals appear larger and swollen whereas in ants they have the shape of a thin cup. Another difference between the two species lies in the vesicle pool within the presynaptic terminals. In ants the LP is densely packed with cVs and only a few dcVs and mitochondria are visible (Schmitt et al., 2016). In the honeybee a higher number of mitochondria and dcVs, but fewer cVs were found. The reason for the differences in the vesicle stock is currently unknown, fixation artifacts seem unlikely due to the high-quality conservation of the tissue.

Microglomerular synaptic complexes of the bulbs in the desert locust *Schistocerca gregaria* share a similar distribution, anatomy and ultrastructure to those in honeybees (Träger et al., 2008). However, one difference arises again in the vesicle stock. In locusts the presynaptic terminal is filled with cVs throughout the profile like in ants, whereas in honeybees vesicles are concentrated near synaptic release sites. Electrophysiological and anatomical studies in locusts showed that these synaptic complexes are part of the sky-compass pathway (Vitzthum et al., 2002; Pfeiffer et al., 2005; Träger et al., 2008). Taken together, the pathway described by Zeller et al. (2015) and the anatomical similarity to locusts shown here strongly suggest that the complexes are part of the sky-compass pathway in honeybees as well.

### Synaptic Complexes in Other Species

In insects, neuromuscular junctions are monads, and most chemical synaptic connections in the central nervous system (CNS) are dyads (Wernitznig et al., 2015). In the visual system, more complex multi-contact synapses have been described in the optic lobes, more precisely in the LA, of muscomorph flies (Shaw and Meinertzhagen, 1986; Meinertzhagen and O’Neil, 1991) and locusts (Wernitznig et al., 2015). In both taxa, photoreceptor neurons provide input to LA monopolar cells via triads and tetrads. At a later stage of visual processing, multi-contact synapses have been mentioned in the calyces of honeybees and in the microglomerular synaptic complexes in the bulbs of the desert locust *Schistocerca gregaria* and in the desert ant *Cataglyphis fortis*. In ants the synaptic connection is formed by triads, tetrads and only a few dyads (Schmitt et al., 2016). Our data revealed a slightly different synaptic formation in the honeybee, with connections being formed by dyads and tetrads. By contrast in locusts the synaptic connections within the microglomerular complexes consists solely of regular ribbons of dyads (Träger et al., 2008). Neither in ants nor in honeybees synapses in the microglomerular complexes are arranged in such a distinguishable and regular manner.

Microglomeruli containing multi-contact synapses also occur in the calyces of the mushroom bodies of insects that are regarded as high-order sensory integration centers. The organization of microglomerular complexes in the calyces is reversed compared to those in the bulbs. In the mushroom body, a microglomerulus consists of one central presynaptic bouton that is surrounded by many postsynaptic profiles belonging to several Kenyon cells (Trujillo-Cenóz and Melamed, 1962; Schürmann, 1974; Ganeshina and Menzel, 2001; Groh and Rössler, 2011). In the complexes in the bulbs of the honeybee it is so far not known if the postsynaptic profiles are related to one or various neurons in one microglomerulus. The calycal microglomerular complexes are smaller than those in the bulbs. In the bulbs of the bee, complexes have a diameter of approximately 8 μm, whereas the size of the microglomeruli in the calyx of honeybees reaches only 2–3 μm (Ganeshina and Menzel, 2001). The synaptic connections in the calyx of honeybees are formed by dyads, triads and tetrads (Groh et al., 2012). In the calycal microglomeruli of fruit flies the number of postsynaptic profiles within one synapse can differ between 1 and 14 (Butcher et al., 2012). Similar to the synaptic complexes in the bulbs, the postsynaptic elements of mushroom body microglomeruli contain high concentrations of motile f-actin (Groh and Rössler, 2011).

Microglomerular synaptic complexes do not only occur in insects. Two well studied types of giant axosomatic synapses in the mammalian CNS, more precisely in the auditory pathway, are the endbulb and the calyx of Held. The presynaptic calyx of Held, probably the largest synaptic terminal in the mammalian CNS, envelopes the soma of a principal cell (Walmsley et al., 1998; review von Gersdorff and Borst, 2002; Schneggenburger and Forsythe, 2006; Rodríguez-Contreras et al., 2008). EM studies in rats showed that one calyx contains about 550 active zones (Sätzler et al., 2002). In comparison, a small glomerulus in the LX of locusts had around 150 active zones (Träger et al., 2008).

### Functional Implications of Microglomerular Synaptic Complexes

Indications for the functional implication of these complexes exist so far only for ring neurons, the *Drosophila melanogaster* equivalent to honeybee TL neurons. There, activity patterns of the dendrites in the bulbs, triggered by a vertical stipe, suggest a retinotopic arrangement and therefore a representative map of the visual surrounding (Seelig and Jayaraman, 2013). While the anatomical data presented in this study provide no direct insight into the physiology of the synaptic complexes of the LX, their structural characteristics allows for some speculations concerning functionality. First, their striking size is remarkable and to our knowledge unique within the insect brain. We assume that the organization and ultrastructure of the complexes leads to a fast and reliable signal transmission. The composition of one large presynaptic terminal enclosing the postsynaptic profiles with all active zones in the center might indicate a low-noise signal transmission. Additionally, the astrocyte-like glial layers around the synaptic complexes likely support reliable transmission. In *Drosophila* astrocyte-like glia is important to clear the synaptic cleft from neurotransmitters and their enzymatic breakdown products (reviewed in Freeman, 2015). In honeybees acetylcholinesterase has been detected in the microglomerular synaptic complexes in the bulbs (Kreissl and Bicker, 1989), suggesting that acetylcholine is likely to act as a transmitter there. NADPH diaphorase labeling in locusts, suggesting nitric oxide synthase activity, revealed staining in TL2 neurons and the LBUs, suggesting the presence of nitric oxide (Kurylas et al., 2005). Nitric oxide is known to function as a retrograde messenger in sensory processing in the nervous system. Since it is gaseous it can pass membranes and diffuse into the surrounding tissue without synaptic release (Dawson and Synder, 1994; Müller, 1997; Bicker, 2001). Therefore, the glia sheaths (GS) around the single complexes might work as diffusion barrier for NO as well as transmitters between adjacent complexes like the ensheathing glia around individual neuropils.

Electrophysiological data of the calyx of Held showed that one single action potential in the presynaptic profile leads to rapid depolarization of the postsynaptic profiles. This on the other hand ensures not only a rapid transmission but also the retention of the timing of signals (Schneggenburger and Forsythe, 2006). Given that the organization of the calyces of Held is comparable to the synaptic complexes in the LX of honeybees, the same principle for fast transmission could be valid here as well. The divergent multi-contact synapses support this assumption, as the transmitter release from one presynaptic membrane simultaneously addresses two or four postsynaptic partners. This could lead to a depolarization of the postsynaptic neuron above threshold by only one presynaptic action potential. So far we could not determine the ratio between the involved pre- and postsynaptic neurons. Whether this ratio is 1:1 as in the calyx of Held, divergent as in locusts, or convergent might be addressed in further studies.

Why do honeybees need such large complexes promoting reliable signal transmission? A closer look at the localization might give some indications. These microglomerular complexes are part of the visual pathway. The preservation of timing is a crucial feature in most sensory pathways to maintain all information of a stimulus. The calyces of Held are part of a pathway for sound-source localization based on time delays, where signal timing is absolutely essential. Another example for the importance of timing for an efficient signal processing is the dual olfactory pathway of the honeybee, where the responses within and between the two tracts reveal an odor-dependent latency (Brill et al., 2013, 2015). Studies on experience-related plasticity of the synaptic complexes in the LX of the desert ant (Schmitt et al., 2016) revealed that the number of synaptic complexes increases upon first exposure to light. The relatively high variation in the total number of synaptic complexes we found in the honeybee may arise from different levels of visual experience in the samples of winter bees used for the present study.

Taken together, the anatomical formation, compared to well-known features of other synaptic complexes, strongly suggests that the microglomerular synaptic complexes in the LX of honeybees and other insects are essential for reliable signal transmission in the sky-compass pathway. It seems plausible that transmission speed and input timing is crucial in a sophisticated visual task like navigation and orientation during flight. However, future neurophysiological experiments on the neurons described here and their synaptic complexes are needed to better understand the properties of signal transmission at this specific point of the visual neuronal system.

## Author Contributions

KP, UH and WR designed the study. MH, AB, CS and RH acquired the data. MH and TSM analyzed and interpreted the data and drafted the manuscript. All authors revised the manuscript critically for important intellectual content, approved the final version to be published and are accountable for all aspects of the work.

## Funding

Funding was provided by the German Research Foundation (DFG) through grant number PF 714/4-1 to KP, and through the Collaborative Research Center SFB 1047 “Insect Timing” (Project B6, WR).

## Conflict of Interest Statement

The authors declare that the research was conducted in the absence of any commercial or financial relationships that could be construed as a potential conflict of interest.

## Footnotes

^1^http://vassarstats.net/

## Abbreviations

AL: antennal lobe
AOT: anterior optic tract
AOTU: anterior optic tubercle
CB: central body
CBL: lower division of the central body
CBU: upper division of the central body
CNS: central nervous system
cV: clear vesicle
CX: central complex
dcV: dense core vesicle
DRA: dorsal rim area
GABA: γ-aminobutyric acid
GS: glia sheath
IT: isthmus tract
KLH: keyhole-limpet hemocyanin
LA: lamina
LBU: lateral bulb
LCA: lateral calyx
LO: lobula
LP: large profile
LUC: lower unit complex of the anterior optic tubercle
LX: lateral complex
M: mitochondrion
MBU: medial bulb
ME: medulla
MEDRA: dorsal rim area of the medulla
N: nucleus
NO: nodulus
NOL: lower division of the nodulus
NOU: upper division of the nodulus
PED: pedunculus
RE: retina
SP: small profile
TL: tangential neuron
TuLAL: tubercle-lateral accessory lobe neuron
UU: upper unit of the anterior optic tubercle
VL: vertical lobe.
